# Distribution of extracellular matrix related proteins in normal and cryptorchid ziwuling black goat testes

**DOI:** 10.1590/1984-3143-AR2022-0005

**Published:** 2022-06-01

**Authors:** Hua Wang, Ligang Yuan, Juanjuan Song, Qianmei Wang, Yong Zhang

**Affiliations:** 1 Gansu Key Laboratory of Animal Generational Physiology and Reproductive Regulation, College of Veterinary Medicine, Gansu Agricultural University, Lan Zhou, Gansu Province, China

**Keywords:** goat, cryptorchid, extracellular matrix, histochemistry, immunofluorescence

## Abstract

The Ziwuling black goat is an indigenously in China, their offspring are frequently affected by congenital cryptorchidism. The extracellular matrix (ECM) contains cytokines and growth factors that regulate the development of the testis, and component changes often result in pathological changes. Cryptorchidism is closely related to structural changes in ECM. In this study, the histochemical staining, immunohistochemical, immunofluorescence and Western blot combined with semi-quantitative analysis was used to describe the distribution of the important ECM components Collagen type IV (Col IV), laminin (LN)and heparan sulfate proteoglycans (HSPG) in the normal and cryptorchid testes of Ziwuling black goats. Results showed that: The histochemical staining showed that the dysplasia of seminiferous tubules and decreased number of Sertoli cells in cryptorchidism, as well as sparse collagen fiber. Meanwhile, the distribution of reticular fibers is relatively rich. Furthermore, the PAS and AB staining in the interstitial vessels and lamina propria of seminiferous tubules is weak. The immunohistochemical and immunofluorescence revealed that Col IV, LN was strongly expressed in Leydig, Sertoli cells of normal testes and moderately positive in the spermatogonia and spermatids, but HSPG was not expressed in the spermatogonia. However, cryptorchidism, the expression of Col IV, LN and HPSG in Leydig, Sertoli cells significantly decreased, as well as the expression of Col IV and LN in capillary endothelial cells, but HSPG was moderately expressed in spermatogonia. Based on these data, the underdevelopment of spermatogenic epithelium, decreased synthesis function of collagen fibers and Leydig cells develop usually in the cryptorchidism were shown to be closely related to the abnormal metabolism of Col IV and LN. The positive expressed of HSPG in the spermatogonia of cryptorchid testes is related to the compensatory development of spermatogonia.

## Introduction

Studies on cryptorchidism, one of the main causes of reproductive infertility in mammals, most cryptorchidism is inherited, but the latest analysis suggests that the environmental factors play a major role in the occurrence and development of cryptorchidism(Xing and Bai, 2018). Generally, the study of cryptorchidism focus on humans and rodents, but reports on cattle, sheep, pigs, and other domestic animals not comprehensive. Insufficient gonadotropin secretion resulting from testicular dysgenesis is one of the most common causes of non-obstructive azoospermia in patients with unilateral or bilateral cryptorchidism (Verkauskas et al., 2019). There are many factors related to cryptorchidism, such as the INSL-3 and androgen both secreted by Leydig cells in the testicular interstitium (Minagawa et al., 2012). INSL3 mainly acts on the fetal period of testicular descent, the fetal testicle can be effectively retained in the groin region (Ivell et al., 2020). The celiac cryptorchidism is basically caused by mutations of INSL3 and receptor (Ivell and Anand-Ivell, 2011), while the inguinal cryptorchidism is mediated by androgen and may have nothing to do with INSL3 (Ivell et al., 2014). We studied that the expression level of INSL3 in inguinal cryptorchidism of Black Goats decreased significantly (Yuan et al., 2021). Inhibition and lack of androgen and its receptors can lead to the inguinal cryptorchidism (França and Godinho, 2003), the expression of androgen receptors in inguinal cryptorchism of Black Goats decreased significantly compared with normal testis (Qianmei et al., 2020).

In addition, the occurrence of cryptorchidism can also cause changes in the testicular extracellular matrix (Yuan et al., 2017a), which is the microenvironment in testicular cells. Therefore, cryptorchidism, as one of the models to study the dynamics of testicular tight junction, is not only used to analyze the regulation of local microenvironment of male reproductive physiology, but also plays an important role in the niche of stem cells, which has been used to cultivate human spermatogonial stem cells, providing a possibility for the treatment of male sterility (Murdock et al., 2019).

Extracellular matrix is synthesized by the gonadal tissue and cells (Wang et al., 2003), which is an important site for the binding of growth factors and cytokines, and involved in organ morphogenesis, cell growth and development regulation, which changes in structure can often lead to corresponding pathological changes in the body (Soito et al., 2011).It contains 102 proteins (Baert et al., 2015), mainly including collagens, laminins (LN), proteoglycan and brake hormone, etc. Therein, the collagens are most abundant components of ECM (Piprek et al., 2018), which are secreted by the interstitial/stromal cells and consistent with the collagen-abundant connective tissue scaffolding (Piprek et al., 2018). LN promotes the development of the spermatic cord during the embryonic period (Heeren et al., 2015). Proteoglycan synthesized by the interstitial/stromal cells may be species-specific (Piprek et al., 2018), which is binding LN, Col IV and other components in the basement and combining with multiple components outside the basement membrane and extracellular multifunctional signal molecules (Jiang et al., 2015). As components of spermatogenic tubule basal membrane and lamina propria, ECM participates in the formation of blood-testosterone barrier and is closely related to testicular development, spermatogenesis and spermatogonial stem cell self-renewal (SSCs) (Zhu et al., 2014).

As demonstrated in a previous study, ECM remodeling in the testicular gubernaculum contributes to testicular descent. In addition, the expression level of LN in cryptorchidism is lower than that in normal testis, which may affect the secretion capacity of Leydig cells (Yuan et al., 2017a).The collagen content in the testes of 15-29 weeks human fetuses and cryptorchid children aged 1.3-10 years significantly decreased, but its fibrogenesis significantly increased (Soito et al., 2011). In vitro studies have revealed that changes in ECM composition of mouse testes can affect morphological changes in Leydig cells, and then affect the secretion of testosterone (Robert B et al., 1991). Furthermore, the expression levels of collagen IV (Col IV) and heparan sulfate proteoglycans (HSPG) in the cryptorchidism of Bactrian camel are significantly lower in Leydig cells than in normal testicular tissue.

The Ziwuling Nature Reserve is a natural gene bank of biological species in China, and its living species are highly representative in the central part of the Loess Plateau (Jia et al., 2019). Ziwuling black goats are highly economically valued for their fur and meat, they are in seasonal estrus, lambing in spring and mainly grow and reproduce in Yulin and Yan’an Cities of Shaanxi Province and Qingyang City of Gansu Province. These animals are characterized by rapid growth, strong adaptability, and stable genetic performance. The Ziwuling black goats initial mating age is 1-1.5 years (Lu et al., 2020), the mating rate is 91.3%~97.5% in normal years, the conception rate is 87.7%~99%, the delivery rate is 87%~90%, and the lambing rate can reach 100%~105% (Huang et al., 2016). According to the investigation, cryptorchidism incidence rate in goats is 0.5%, in long-term inbred sheep; its incidence rate often can be as high as 10% or more. In this study, the histochemical characteristics of the components of ECM and distribution of Col IV, LN and HSPG in normal testes and cryptorchid testes of Ziwuling black goats were compared and analyzed by immunohistochemical staining, immunohistochemical and immunofluorescence techniques, to provide morphological reference for the study of testicular pathology and reproductive physiology of Ziwuling black goats.

## Methods

### Animals and tissue preparation

There were 12 pilot samples, 6 normal testes and 6 cryptorchidism, were surgically removed from 5-month-old Ziwuling black goats in Huanxian Pastoral Area, Qingyang City, Gansu Province, and divided into 2 groups. One sample was frozen in liquid nitrogen for western blot, and the other group was fixed in 4% formaldehyde for later use. The animal procedures used in this study were reviewed and approved by the Gansu Agricultural University’s Academic Committee and the National Natural Science Foundation of China according to guidelines established by the Biological Studies Animal Care and Use Committee of Gansu Province (Approval No. 31660670).

### Testicular tissue sample preparation

Sections were prepared as described in our previous study (Li et al., 2016). Briefly, the testicular tissues were fixed in 4% formaldehyde, dehydrated in different ethyl alcohol concentrations, trans parented in xylene, and then embedded in paraffin. Each sample was sliced continuously to produce 24 groups (6 pieces in each group).

Hematoxylin and eosin (H&E), Masson collagen fiber, Gomori silver ammonia, Alcian blue (AB, pH=2. 5), periodic acid-Schiff staining (PAS), and Alcian blue-periodic acid-Schiff staining (AB-PAS, pH=2. 5), staining methods were employed to analyze the histochemical characteristics of normal and cryptorchid testes of Ziwuling black goats.

### 2.3 Immunohistochemistry (IHC) assay

Tissue sections were dewaxed and rehydrated in xylene and distilled water. The endogenous peroxidase was blocked with 3% hydrogen peroxide in the tissue sections at 37°C for 15 min. Then, goat serum albumin was incubated with 5% for 15 min and subsequently incubated with rabbit anti-Col IV (0.33*10^-2^mg/ml, bs-0806R; RRID: AB_10855678; Bioss, Beijing, China), rabbit Anti-LN (0.33*10^-2^mg/ml, bs-0821R, RRID: AB_10856798, Bioss), and rabbit anti-perlecan/HSPG (0.33*10^-2^mg/ml, bs-5072R, RRID: AB_10856731, Bioss) polyclonal antibodies overnight at 4°C in a humidified chamber. Following incubation, the sections were washed with phosphate buffered saline (PBS), incubated with a biotin-labeled secondary antibody (SP Kit [rabbit], SP-0023, Bioss) for 15 min, washed again with PBS. Afterwards, incubated with the streptavidin peroxidase component from an immunohistochemical kit (Solarbio, Shanghai, China) for15 min, added the HRP-DAB chromogenic kit (Solarbio, Shanghai, China). Finally, the sections were counterstained with hematoxylin, rinsed with tap water, and observed under a microscope (DP73, Olympus; Tokyo, Japan).

### Immunofluorescence (IF) assay

After the biotin-labeled secondary antibody was replaced with goat anti-rabbit IgG (0.125*10^-2^mg/ml, bs-0259G-AF488, Bioss), and incubated at 4°C for 1 hr in a cassette. The rest of the programs were the same at the operating procedures of IHC. In the end, immunofluorescence images were collected by a microscope (RVL100-G, ECHO, USA).

### IF double-staining

Biotin-labeled secondary antibody was replaced with 0.125*10^-2^mg/ml diluted goat anti-rabbit IgG. Thereafter, the sections were incubated at 4°C for 45 min. The rest of the programs were the same at the operating procedures of IHC. Then incubated 10 min with DAPI (C02-04002, Bioss) in cassette and washed with PBS. Lastly, the photographs were collected by a microscope (LSM 800; Carl Zeiss, Germany).

### Western blot

The total protein was extracted using total protein extraction kit (TransGen Biotech Co., Beijing, China), confirmed by western blot using primary antibody Col IV, LN and HSPG (0.01*10^-2^mg/ml), (Abcam, USA) at 4°C for 12h. After that, PVDF (TransGen Biotech Co., Beijing, China) was incubated in rabbit IgG was used secondary antibody at 37°C for 2h, as well as was used for exposure in the darkroom; β-actin was used as the internal reference.

### Statistical analysis

5 slices were selected for each stain randomly, and 6 non-repetitive fields were picked from each slice (×400). Statistical analyses were performed by the SPSS statistical software program (SPSS, RRID: SCR_002865). The data were presented as mean ± SEM, and the differences were regarded as significant at p < 0.05.

Images were analyzed by using Image J 8.0 analysis software (National Center for Microscopy and Imaging Research: Image J Mosaic Plug-ins, RRID: SCR_001935) to assess the seminiferous tubule characteristic indexes, such as the number of supporting cells and ratio of interstitial area to the average seminiferous tubule. In addition, the images were analyzed by the Image-Pro plus 6.0 software (Image-Pro Plus, RRID: SCR_007369) was used to detect the fluorescence intensity.

## Results

### Comparison of histochemical characteristics of the testes in normal testes and cryptorchid testes of Ziwuling black goats

**Figure 1:**
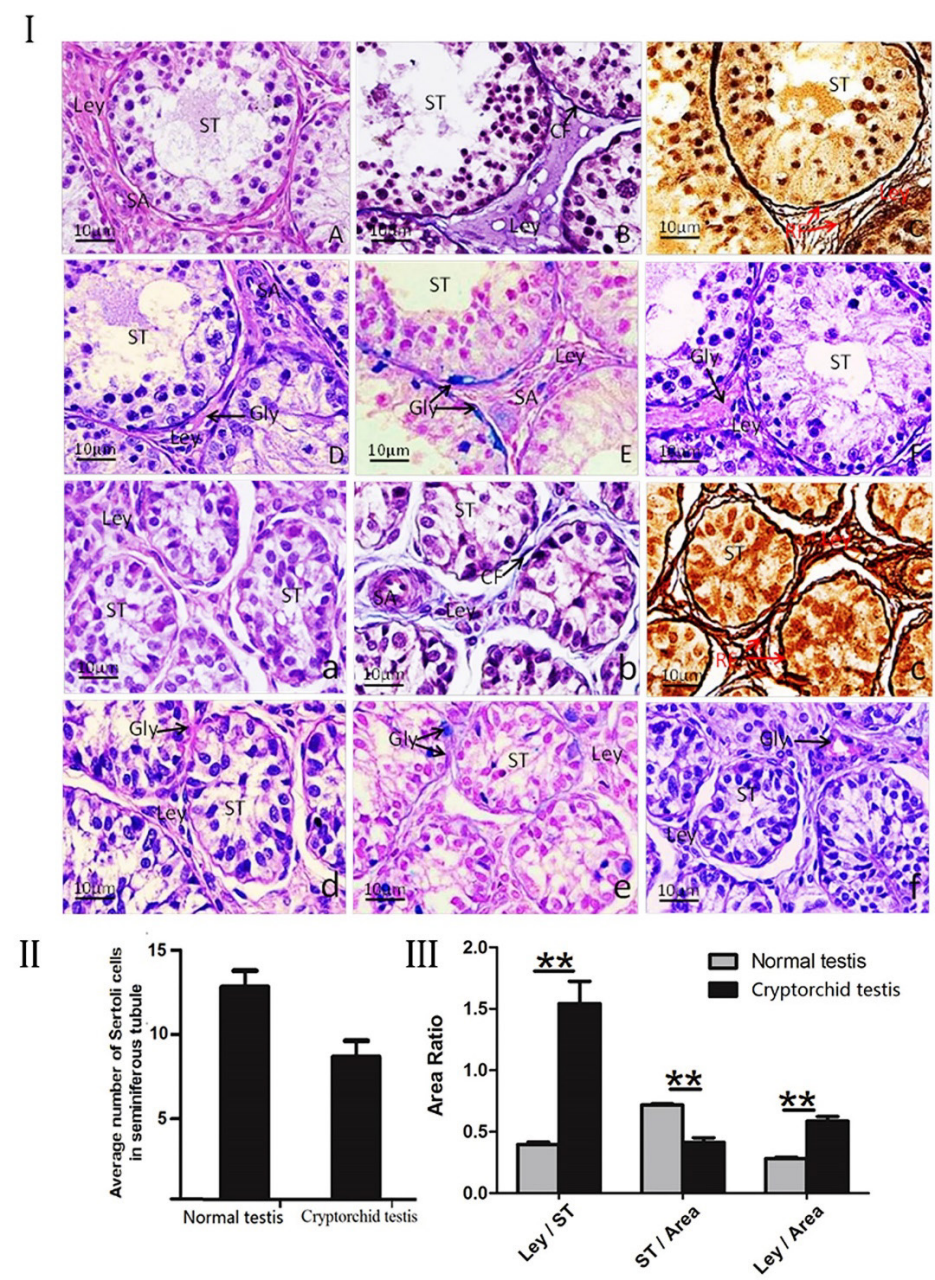
(I). Comparison of the histochemical characteristics between the normal and the cryptorchid testis of Ziwuling black goats. A-F: Normal testis; a-f: cryptorchid testis; (A, a): H&E staining; (B, b): Masson staining; (C, c): Gomori silver ammonia staining; (D, d) PAS staining; (E, e): AB staining; (F, f) AB-PAS staining; A-C: Revealed that the seminiferous epithelium of normal testis was developing well in the normal group; (D): The positive PAS reactions were observed at the attachment of spermatogenic epithelial sperm cells clearly; (E): The AB staining blue positive bands were clearly and obvious in the lamina propria; (F): AB-PAS showed obvious positive reaction in the interstitial tissue and lamina propria of the seminiferous tubules; (a-c): The cryptorchidism causes a reduction in layers of spermatogenic epithelium; (d): Decreased PAS positive in the basement membrane of the seminiferous tubule; (e): The positive reaction of AB was almost invisible in the interstitial tissue; (f): AB-PAS staining shown a weak positive reaction in the interstitial tissue and lamina propria of the seminiferous tubules. Magnification: 1000×, scale bar=10μm. ST: seminiferious tubules; CF: Collagen fiber; Gly: Glycogen; Ley: leydig cell; Sc: Sertoli cells; Small artery (SA); (II), the area ratio of interstitial tissue to seminiferous tubule; **p < 0. 01; (III); the average number of Sertoil cells in seminiferous tubule between normal testis and cryptorchid testis. All graphs show mean ± scanning electron microscope (SEM) from three independent experiments.

Under the light microscope, the normal testicular interstitial connective tissues of Ziwuling black goats were obvious. Spermatogenic epithelium consisted of 3-5 layers of spermatogenic cells, columnar Sertoli cells, the spermatozoa were obviously distributed in the lumen, and myoid cells with long cord-shaped nuclei were observed surrounding the lamina propria of seminiferous tubules (Figure 1I. A). Collagen fibers were sparsely distributed around the lamina propria and periphery of small vessels of the seminiferous tubules, Leydig cells were scattered among the collagen fibers (Figure 1I. B). Abundant reticular fibers were also found in the lamina propria of the seminiferous tubules and the peripheral basement membrane of the interstitial vessels (Figure 1I. C). PAS staining red glycogen positive bands were found in the lamina propria and the interstitial capillary walls. In addition, positive PAS reactions were also observed at the attachment of spermatogenic epithelial sperm cells. (Figure 1I. D); AB staining blue positive bands were clearly and obvious in the lamina propria (Figure 1I. E); AB-PAS showed obvious positive reaction in the interstitial tissue and lamina propria of the seminiferous tubules (Figure 1I. F).

However, compared with the normal group, in cryptorchid testes, lymphatic capillaries and blood vessels of interstitial tissue testes were scattered between the connective tissues. The seminiferous epithelium was composed of 2-3 layers of cells, and Sertoli cells were the most numerous. Leydig nuclei were scattered among the connective tissues (Figure 1I. a). Collagen fibers were sparsely distributed around the periphery of seminiferous tubules (Figure 1I. b). And the distribution of reticular fibers was obvious in the lamina propria and interstitial tissue of the seminiferous tubules (Figure 1I. c), PAS staining positive reactions were found in the interstitial tissue (Figure 1I. d). AB staining did not reveal significant blue positive bands in the interstitial tissue (Figure 1I. e). AB-PAS staining shown a weak positive reaction in the interstitial tissue and lamina propria of the seminiferous tubules (Figure 1I. f).

### Comparison of characteristic components of seminiferous tubules in normal and cryptorchid testes of Ziwuling black goats

Statistical analysis revealed that the number of Sertoli cells in cryptorchidism was significantly increased, the mean diameter of seminiferous tubules was decreased evidently in cryptorchid testes of Ziwuling black goat compared with normal group (P<0. 05, Table 1, Figure 1II, III), the interstitial tissue surface was positively and significantly increased, and the interstitial/lumen area was extremely significantly increased compared with the normal group. (p<0.01, Table 1, Figure 1. III).

**Table 1:** Comparison of spermatogenic tubule index between testes and cryptorchidism (n=30).

	**Numbers of Sertoli cells (n)**	**Diameter of the seminiferous tubules (μm)**	**Bureaucratic area (μm^2^)**	**Interstitial area (μm^2^)**	**Ratio of interstitial area and bureaucratic area**
The scrotal testis of goat	55.4 ± 3.8	84. 43 ± 15.67	21244.75± 133.83	3798.76 ± 123.88	0.178 ±0.32
The cryptorchid testis of goat	89.7 ± 5.2*	47. 279 ±11. 82*	24897.55 ± 114.67**	6448.39 ± 140.68**	0.259 ± 0.041*

In the same column, *, Significant difference (P<0. 05);**, Extremely significant difference (P<0.01) .

### Comparison of ECM related proteins distribution in normal and cryptorchid testes of Ziwuling black goats

**Figure 2:**
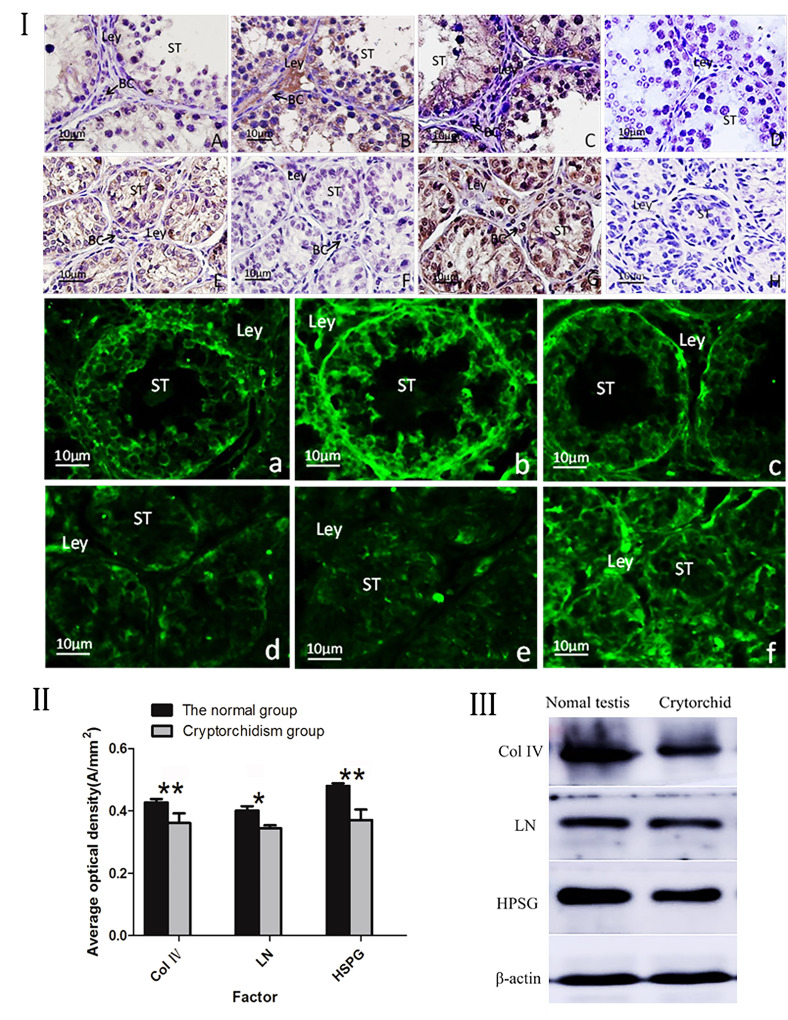
(I). IHC and IF assay of ECM related proteins in normal and cryptorchid testis of Ziwuling black goats. A-D: IHC assay the normal testis; E-H: IHC assay the cryptorchid testis, a-c: IF assay the normal testis; d-f: IF assay the cryptorchid testis. Magnification: 400 ×, scale bar=20μm. (A, a): The positive expression of Col IV in the Leydig cells and basal membrane of the seminiferous tubules was significantly, but not in the spermatogonia; (B, b): LN was strongly positive in the Sertoli cells, Leydig cells, peritubular myoid cells spermatids and subbasement capillaries; (C, c): HSPG was strongly positive in the Leydig and Sertoli cells, but not in spermatogonia and weakly expressed in other spermatogenic cells; (E, d): Col IV was weakly expressed in Leydig cells and seminiferous epithelium, but not in Sertoli cells and spermatogonia; (F. e): LN was strongly positive in the Sertoli, Leydig and peritubular myoid cells, and moderately positive in the spermatogonia and capillary wall cells; (G, f): HSPG was strongly positive in spermatogonia, moderately positive in spermatids, and weakly expressed in the Sertoli, Leydig, peritubular myoid cells and capillaries; D and H: negative control (no significant immunoreactivity was observed when normal rabbit serum instead of the primary antibody). Green color: immunofluorescence representing the reaction of antibodies with antigens. BC: Blood capillary; Ley: Leydig cells; pc: Peritubular myoid cells; Sc: Sertoli cells; SG: Spermatogonia; ST: seminiferious tubule. (II). The average optical density of Col IV, LN and HSPG is between normal and cryptorchid testis. n= 12;*p< 0.05; **p < 0.01. (III) Western blot analysis of Col IV, LN, HSPG expression in normal and cryptorchid testes in Ziwuling black goats.

Immunohistochemical analysis of ECM related proteins in normal testes showed that the positive expression of Col IV in the Leydig cells and basement membrane of the seminiferous tubules was significantly, but not in the spermatogonia (Figure 2I. A). LN was strongly positive in the Sertoli cells, Leydig cells, peritubular myoid cells and sub-basement capillaries, particularly in Sertoli cells, as well as in spermatids (Figure 2I. B). HSPG was strongly positive in the Leydig cells and Sertoli cells, but not in spermatogonia and weakly expressed in other spermatogenic cells (Figure 2I. C).

Immunohistochemical analysis of ECM related proteins in cryptorchidism showed that Col IV was weakly expressed in Leydig cells and seminiferous epithelium, but not in Sertoli cells and spermatogonia (Figure 2I. E). LN was strongly positive in the sub-basement capillaries and moderately positive in Sertoli cells, Leydig cells and the peritubular myoid cells, as well as expressed in spermatogonia and spermatid (Figure 2I. F). HSPG was strongly positive in spermatogonia, moderately positive in spermatids, and weakly expressed in the Sertoli cell, Leydig cells, peritubular myoid cells and capillaries (Figure 2I. G). No positive expression in the negative control group (Figure 2I. D and H).

Expression levels of Col IV, LN and HSPG were significantly lower in normal testes than that in cryptorchid (*p < 0.05; **p < 0.01) (Figure 2II); The Western blot showed that the Col IV, LN and HSPG expression were observed in both normal and cryptorchid, which were same as Immunohistochemical analysis (Figure 2III).

### Comparison of ECM related protein localization in normal and cryptorchid testes of Ziwuling black goats

IF test results of normal testicular ECM related proteins (Table 2) showed that Col IV was high density and strongly positive in Sertoli cells, Leydig cells and capillary wall cells, strongly positive in the peritubular myoid cells, and moderately positive in the spermatogonia (Figure 2I. a). LN was strongly positive in the Sertoli, Leydig and peritubular myoid cells, and moderately positive in the spermatogonia and capillary wall cells (Figure 2I. b). HSPG was high-density and strongly positive in Leydig cells, strongly positive in spermatid and peritubular myoid cells, and moderately positive in the Sertoli cells, but no positive expression was found in spermatogonia (Figure 2I. c).

**Table 2:** **T**he distribute of Col IV, LN and HSPG in different parts of the normal and cryptorchid testis of Ziwuling black goats.

**Group**	**Sertoli cells**	**Spermatogonia**	**Spermatid**	**Leydig cells**	**Peritubular myoid cells**	**Endothelial cells**
Col IV-The scrotal group	+ + +	+	+	+ + +	+ +	+ + +
Col IV-The cryptorchid group	+	-	+	+	+ +	+
LN-The scrotal group	+ + +	+	+ +	+ + +	+ + +	+ +
LN-The cryptorchid group	-	+	-	+ +	+	+ +
HSPG-The scrotal group	+ +	-	+ +	+ + +	+ +	+
HSPG-The cryptorchid group	+ +	+ + +	+	+ +	+ +	+

In cryptorchid testes, Col IV was positively expressed in Sertoli cells, Leydig cells and capillary wall cells, but not in spermatogonia (Figure 2I. d). LN was not expressed in Sertoli cells and spermatids, but was moderately positive in Leydig cells and capillary wall cells, and positive in spermatogonia and peritubular myoid cells (Figure 2I. e). HSPG was strongly positive in the spermatogonia, moderately positive in Sertoli cells and Leydig cells, and positive in spermatid and capillary wall cells (Figure 2I. f).

### Comparison of Col IV, LN and HSPG dual location protein in normal and cryptorchid testes of Ziwuling black goats

**Figure 3:**
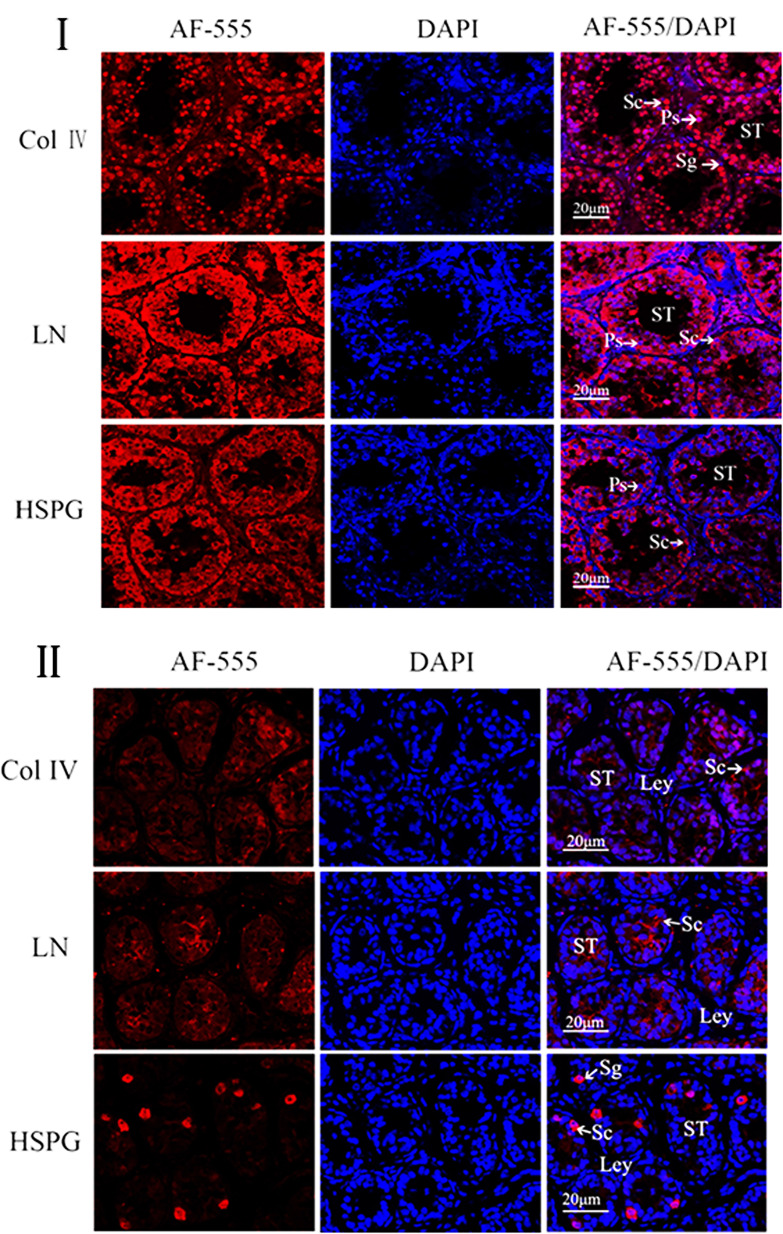
Immunofluorescence doubles staining of ECM-associated proteins in normal and cryptorchid testis. (I) Immunofluorescence doubles staining of the normal testis. (II) Immunofluorescence doubles staining of the cryptorchid testis. Magnification: 400×. Blue color: DAPI-stained nuclei; Red color: immunofluorescence representing the reaction of antibodies with antigens. Leydig cells (Ley); Sertoli cells (Sc); Seminiferous tubules (ST); Primary spermatocytes (Ps). Scale bar=20μm

The comparative analysis on the expression results of normal and cryptorchid testes showed that the expression levels of Col IV and LN were significantly decreased in cryptorchid testes, mainly manifested by the decrease in the expression of Leydig cells and Sertoli cells; the expression of HSPG was relatively obvious in the Sertoli cells of normal testes, while in cryptorchid testes, it mainly expressed in spermatogonia (Figure 3I, Figure 3II).

## Discussion

### Comparison of interstitial tissue in normal and cryptorchid testes of Ziwuling black goats

The connective tissue of mammalian testicular interstitial is the scaffold of interstitial blood vessels, lymphatics and Leydig cells. The change of interstitial connective tissue may affect the local nutrient metabolism of testicular interstitial tissue. For instance, to some extent, an increase of interstitial connective tissue in bovine testes can cause the decrease of sperm quality (Hoflack et al., 2008). A prior study has revealed that seminiferous tubule dysgenesis further loosens the intratubular connective tissue in cryptorchid Bactrian camels (Yuan et al., 2016). Studies on the testes of ruminants in plateau areas suggest that interstitial connective tissue may influence the development of seminiferous epithelium to some extent (Yuan et al., 2017a). This study is consistent with the above research, the average diameter of seminiferous tubules in cryptorchidism of Ziwuling black goats is significantly smaller than that in the normal group, the interstitial collagen fibers are sparse, and the interstitial/lumen area is extremely significantly larger than that in the normal group. According to relevant studies, the increase of interstitial area will adversely affect the quantity and quality of sperm, resulting in the decline of spermatogenic function (Pitia et al., 2017). Consistent with this study, the changes of cryptorchid interstitial tissue of Ziwuling black goats can be one of the factors of spermatogenesis decline.

ECM is the basic component that mediates interaction between Leydig cells and peritubule cells, including the basal membrane of seminiferous tubules and the matrix of peritubular cells. As previously mentioned, peritubular myoid cells not only maintain the structural integrity of seminiferous tubules, but also participate in spermatogenesis and regulation of testicular function. Studies have reported that the reticular fibers around the seminiferous tubules in rats can provide support the structure of reproductive epithelium, and experimental varicocele induces hypoxia is frequently accompanied by some degree of testicular fibrosis (Gur et al., 2021). The collagen protein, elastin, proteoglycan and glycoprotein are synthesized by fibroblasts cells and form fibers matrix (Jia et al., 2018). In this study, the low content of collagen fibers in the interstitial tissue of cryptorchid testes may be caused by decreased secretory capacity of fibroblasts. Ezeasor (1985) reported that the loosened connective tissue between the seminiferous tubules may be caused by the dysgenesis of seminiferous tubules in goats cryptorchidism (Yuan et al., 2021). Therefore, in this study, the sparse distribution of interstitial collagen fibers may be related to the dysgenesis of seminiferous tubule in cryptorchid goats.

The positive reaction of glycogen, glycoprotein and proteoglycan in PAS, AB and AB-PAS staining is typically exist in the connective tissue, mucus, and basement membrane of the testes. Previous studies have shown that PAS positive reaction is mainly located in the connective tissue, basement membrane of seminiferous tubules and Leydig cells in rat testes. The PAS positive reaction was enhanced as spermatogenic cells matured. It is increased during the early reproductive cycle, and decreased significantly in the late stage (Fayomi and Orwig, 2018). Meanwhile, the positive reaction of PAS and AB-PAS have been detected in the interstitial blood vessels and the lamina propria of seminiferous tubules in testicular tissues of Bactrian camels and dromedaries. In line with this, carbohydrate is essential for spermatogenesis during the development of the reproductive epithelium and can be provided to the epithelium through blood vessels and lamina propria of seminiferous tubules (Yuan et al., 2017b). It is reported that the positive reaction of AB-PAS in testes of alligators is mainly located in the connective tissue, Leydig cells and basement membrane of seminiferous tubules, and it was enhanced with the maturation of the spermatogenic cells (Moore et al., 2010). Compare with the normal testes of Bactrian camel, the decrease of positive reaction with PAS and AB-PAS in cryptorchid testes may be related to abnormal development of seminiferous tubule and the decrease of connective tissue synthesis in interstitial tissue (Yuan et al., 2017a). Therefore, in this study, PAS positive reaction, AB positive reaction and AB-PAS positive reaction in cryptorchid testes are significantly reduced compared with normal testes, which may be related to the developmental changes of seminiferous tubules and interstitial tissues. AB (pH=2. 5) staining is mainly used to distinguish sulfated acid mucin from neutral mucin. Previous studies have shown that the sulfated acid mucin produced by Sertoli cells may be involved in the cytoskeleton of sperm tail formation and complement defense, etc. During the maturation of Sertoli cells, sulfated acid mucins secreted by Sertoli cells may influence their morphological differentiation and maturation, and also play a role in spermatogenesis and regulation after puberty (Qin et al., 2021), are consistent with this study, the AB reaction in the basement membrane of seminiferous tubules is strongly positive in normal testes, while there is almost no positive reaction in cryptorchid testes. The results indicated that the content of sulfate acid mucin decreased sharply during cryptorchidism, suggested that neutral mucin is the major mucin in the basement membrane of seminiferous tubules.

### Comparison of ECM related protein distribution in normal and cryptorchid testes of Ziwuling black goats

ECM components of testes are mainly produced by Sertoli cells in seminiferous tubules and peritubular myoid cells in the lamina propria, both of which cooperate with each other in the formation of ECM. Generally, Sertoli cells generate Col IV, LN, actin and HSPG, while the peritubular myoid cells synthesizes LN, ColI, and Col IV (Park et al., 2016). Studies have shown that the important components of testicular ECM, such as Col IV, LN and HSPG, interact with proteases and protease inhibitors of spermatogenic cells, TNFα and other cytokines to mutually regulate spermatogenesis (Siu and Cheng, 2008). Col IV forms a structural network, which exists in the basement membrane of all organs, and is the major collagen component in the basement membrane of seminiferous tubules. It is also involved in the formation of Sertoli cell tight junctions and the dynamic regulation of the blood-testis barrier during spermatogenesis (Li et al., 2020). Col IV mainly exists in basement membrane, Sertoli cells, spermatogenic cells and myoid cells in bovine testes (Berkholtz et al., 2006). In vitro studies indicates that adding Col IV in mediums is beneficial to the growth and differentiation of porcine SSCS (Zhao et al., 2018). Consistent with the mentioned result, Col IV is positively expressed in spermatogenic cells and peritubular myoid cells, and strong positively expressed in Sertoli cells, Leydig cells and capillary wall cells in normal testicular tissues of Ziwuling black goats. Therefore, in this study, the expression level of Col IV in Sertoli cells, Leydig cells and peritubular myoid cells in cryptorchid tissue of Ziwuling black goats is significantly reduced. It means that Sertoli cells and peritubular myoid cells are closely related to the synthesis and secretion of Col IV. In addition, spermatogenesis is not only regulated by hormones secreted from the hypothalamo-pituitary-gonadal axis, but also depends on paracrine signaling to regulate the physiological function of testes. Normal spermatogenesis depends on testosterone, and paracrine between peritubular cells and Sertoli cells is involved in regulating testosterone secretion by Leydig cells (Hafizuddin et al., 2020). In testes, only Leydig cells, Sertoli cells and peritubular cells express androgen receptor (AR), but there is no androgen receptor on mature spermatogenic cells (Walker, 2009). Hence, testosterone, as a major androgen, does not act on spermatogenic cells directly, instead indirectly through Sertoli cells. Thus, the expression of Col IV in cryptorchid testes of Ziwuling black goats significantly decreased is intimately associated with the function decline of Col IV secretion and synthesis of Leydig cells, Sertoli cells and peritubular myoid cells, which caused by their morphological changes. On the other hand, the decrease of Col IV secretion may be related to the compensatory maintenance of testosterone secretion by Leydig cells during cryptorchidism. This result indicated that Sertoli cells and peritubular myoid cells are strongly associated with synthesis of Col IV.

LN is widespread in the matrix, and it is the most abundant ECM component in the basement membrane, which has many linking and mediating effects to controll the formation of basement membrane (Binsila et al., 2018; Park et al., 2016). Cooperation between Sertoli cells and peritubular myoid cells contribute to the secretion of LN. Col IV and LN are distributed along the epithelial basement membrane and around peritubular myoid and Sertoli cells, and the gradual differentiation of Sertoli cells and peritubular cells are associated with the biosynthesis of peritubular matrix (Tung and Fritz, 1980). In addition, in vitro studies have shown that Sertoli cells are observed to respond to LN by increasing calcium concentration in rat testicular tissue, and LN may modulate Sertoli cells by disrupting intracellular calcium homeostasis (Taranta et al., 2000). A previous study has revealed that the immunolocalization of LN in the testes of adult black-backed jackals is located to the basement membrane around the seminiferous tubules, Leydig cells and peritubular myoid cells, particularly strongly expressed in peritubular myoid and Sertoli cells (Madekurozwa and Booyse, 2017). Similarly, the results of this study showed that Col IV and LN positive immunohistochemical staining of normal testes are mainly located in the basement membrane and distributed along the peritubular myoid and Sertoli cells. Therefore, peritubular myoid cells and Sertoli cells may be involved in the synthesis and secretion of Col IV and LN. Studies have reported that LN is obviously distributed in the lamina propria of seminiferous tubules in infants and adult males with cryptorchidism, but not in Sertoli cells or spermatid (Santamaria et al., 1990). In this study, there is no positive expression in Sertoli cells and spermatid of cryptorchid Ziwuling black goats, while weak positive expression is observed in peritubular myoid and Leydig cells. This finding suggests that LN is directly associated with the development and maturation of Sertoli cells and spermatid.

HSPG is an important component commonly present in the basement membrane of human spermatogenic epithelium. It plays an essential role not only in binding LN, Col IV and other components in the basement membrane, but also in combining with multiple components outside the basement membrane and extracellular multifunctional signal molecules (Jiang et al., 2015). In vitro studies have shown that HSPG is involved in the regulation of testosterone production in Leydig cells of adult rats (McFarlane et al., 1996). However, HSPG is not expressed in mature and highly differentiated epithelial cells (Hayashi et al., 1987). Related studies have also shown that distribution of HSPG in testes of aged yaks is significantly lower than that of young yaks, and the expression of Sertoli cells does not change significantly, while the expression of Leydig cells decreased, so HSPG may indirectly affect the testosterone secretion by Leydig cells (Yuan et al., 2015). HSPG is not found expressed in spermatogonia in normal testes of Bactrian camels (Yuan et al., 2016), but is relatively strongly expressed in spermatogonia in cryptorchid testes, and is significantly expressed in spermatid and spermatocyte epithelium. Hence, it can be concluded that HSPG is closely associated with spermatogonia development (Hayashi et al., 1987). In a recent study (Yuan et al., 2017a), HSPG is shown to be associated with Sertoli cells development in rats. In this study, the expression level of HSPG in the spermatogonia of normal testes group is significantly lower than that of cryptorchid testes group, indicating that the spermatogonia of Ziwuling black goats is immature development during cryptorchidism, which may be one of the reasons for the significant increase of HSPG expression level in spermatogenic epithelium.

## Conclusion

In cryptorchid Ziwuling black goats, seminiferous tubules hypoplasia, significantly increased of interstitial tissue and decreased synthesis ability of collagen fibers, as well as significant basement membrane hyperplasia of the seminiferous epithelium, which affect the sugar and other nutrients (e.g., carbohydrate) supplied to the epithelium through blood vessels and seminiferous tubule lamina propria. In addition, there is a significant difference in the important components of ECM (e.g., Col IV and LN) between normal and cryptorchid testes of Ziwuling black goat, which affects the normal development of seminiferous tubules and Leydig cells. A strong positive expression of HSPG in the seminiferous epithelium of cryptorchid Ziwuling black goats is identified to be associated with the immature development of spermatogonia.

## References

[B001] Baert Y, Stukenborg J-B, Landreh M, De Kock J, Jornvall H, Soder O, Goossens E (2015). Derivation and characterization of a cytocompatible scaffold from human testis. Hum Reprod.

[B002] Berkholtz CB, Lai BE, Woodruff TK, Shea LD (2006). Distribution of extracellular matrix proteins type I collagen, type IV collagen, fibronectin, and laminin in mouse folliculogenesis. Histochem Cell Biol.

[B003] Binsila KB, Selvaraju S, Ghosh SK, Parthipan S, Archana SS, Arangasamy A, Prasad JK, Bhatta R, Ravindra JP (2018). Isolation and enrichment of putative spermatogonial stem cells from ram (Ovis aries) testis. Anim Reprod Sci.

[B004] Ezeasor D (1985). Light and electron microscopical observations on the Leydig cells of the scrotal and abdominaltestes of naturally unilateral cryptorchid West African dwarf goats. J Anat.

[B005] Fayomi AP, Orwig KE (2018). Spermatogonial stem cells and spermatogenesis in mice, monkeys and men. Stem Cell Res.

[B006] França LR, Godinho CL (2003). Testis morphometry, seminiferous epithelium cycle length, and daily sperm production in domestic cats (Felis catus). Biol Reprod.

[B007] Gur FM, Timurkaan S, Taskin E, Guven C, Gur HE, Senturk M, Dastan S, Nurdinov N, Unalan A, Cankut S, Tatyuz I (2021). Thymoquinone improves testicular damage and sperm quality in experimentally varicocele‐induced adolescent rats. Andrologia.

[B008] Hafizuddin H, Karja NWK, Praharani L, Setiadi MA (2020). Adiponectin and testosterone levels and their correlations with fertility in Anglo-Nubian x Etawah Grade Crossbred Bucks. Trop Anim Sci J.

[B009] Hayashi K, Hayashi M, Jalkanen M, Firestone JH, Trelstad RL, Bernfield M (1987). Immunocytochemistry of cell surface heparan sulfate proteoglycan in mouse tissues. A light and electron microscopic study. J Histochem Cytochem.

[B010] Heeren AM, van Iperen L, Klootwijk DB, de Melo Bernardo A, Roost MS, Gomes Fernandes MM, Louwe LA, Hilders CG, Helmerhorst FM, van der Westerlaken LA, Chuva de Sousa Lopes SM (2015). Development of the follicular basement membrane during human gametogenesis and early folliculogenesis. BMC Dev Biol.

[B011] Hoflack G, Van den Broeck W, Maes D, Van Damme K, Opsomer G, Duchateau L, de Kruif A, Rodriguez-Martinez H, Van Soom A (2008). Testicular dysfunction is responsible for low sperm quality in Belgian Blue bulls. Theriogenology.

[B012] Huang Y-F, Chen L-P, Zhao Y-J, Zhang H, Na R-S, Zhao Z-Q, Zhang JH, Jiang CD, Ma YH, Sun YW, e GX (2016). Complete mitochondrial genome of Chuanzhong black goat in southwest of China (Capra hircus). Mitochondrial DNA A DNA Mapp Seq Anal.

[B013] Ivell R, Alhujaili W, Kohsaka T, Anand-Ivell R (2020). Physiology and evolution of the INSL3/RXFP2 hormone/receptor system in higher vertebrates. Gen Comp Endocrinol.

[B014] Ivell R, Anand-Ivell R (2011). Biological role and clinical significance of insulin-like peptide 3. Curr Opin Endocrinol Diabetes Obes.

[B015] Ivell R, Heng K, Anand-Ivell R (2014). Insulin-like factor 3 and the HPG axis in the male. Front Endocrinol (Lausanne).

[B016] Jia J, Han L, Wang X, Liu W (2019). Risk and regionalization of drought for Winter Wheat in Gansu Province. Arid Zo Res.

[B017] Jia Y, Zhou J, Chang Y, An F, Li XW, Xu XY, Sun XL, Xiong CY, Wang JL (2018). Effect of optimized concentrations of basic fibroblast growth factor and epidermal growth factor on proliferation of fibroblasts and expression of collagen: related to pelvic floor tissue regeneration. Chin Med J (Engl).

[B018] Jiang D, Fu X, Sheng Z (2015). The diversity of structure and function of heparin sulfate proteoglycans via modification of some relative enzymes. Chinese J Pathophysiol.

[B019] Li C, Yuan L, Zhang Y (2016). The distribution of extracellular matrix proteins in small-tail han sheep epididymis in Plateau. Chinese J Anim Vet Sci.

[B020] Li L, Zhang L, Zhang Z, Keyhani NO, Xin Q, Miao Z, Zhu Z, Wang Z, Qiu J, Zheng N (2020). Comparative transcriptome and histomorphology analysis of testis tissues from mulard and Pekin ducks. Arch Tierzucht.

[B021] Lu T, Zou X, Liu G, Deng M, Sun B, Guo Y, Liu D, Li Y (2020). A preliminary study on the characteristics of micrornas in ovarian stroma and follicles of chuanzhong black goat during estrus. Genes (Basel).

[B022] Madekurozwa MC, Booyse D (2017). Seasonal changes in the immunolocalization of cytoskeletal proteins and laminin in the testis of the black-backed jackal (Canis mesomelas). J Vet Med Ser C Anat Histol Embryol.

[B023] McFarlane JR, Laslett A, De Kretser DM, Risbridger GP (1996). Evidence that heparin binding autocrine factors modulate testosterone production by the adult rat Leydig cell. Mol Cell Endocrinol.

[B024] Minagawa I, Fukuda M, Ishige H, Kohriki H, Shibata M, Park EY, Kawarasaki T, Kohsaka T (2012). Relaxin-like factor (RLF)/insulin-like peptide 3 (INSL3) is secreted from testicular Leydig cells as a monomeric protein comprising three domains B–C–A with full biological activity in boars. Biochem J.

[B025] Moore BC, Hamlin HJ, Botteri NL, Lawler AN, Mathavan KK, Guillette LJ (2010). Posthatching development of Alligator mississippiensis ovary and testis. J Morphol.

[B026] Murdock MH, David S, Swinehart IT, Reing JE, Tran K, Gassei K, Orwig KE, Badylak SF (2019). Human testis extracellular matrix enhances human spermatogonial stem cell survival in vitro. Tissue Eng Part A.

[B027] Park MH, Park JE, Kim MS, Lee KY, Hwang JY, Yun JI, Choi JH, Lee E, Lee ST (2016). Effects of extracellular matrix protein-derived signaling on the maintenance of the undifferentiated state of spermatogonial stem cells from porcine neonatal testis. Asian-Australas J Anim Sci.

[B028] Piprek RP, Kolasa M, Podkowa D, Kloc M, Kubiak JZ (2018). Transcriptional profiling validates involvement of extracellular matrix and proteinases genes in mouse gonad development. Mech Dev.

[B029] Pitia AM, Uchiyama K, Sano H, Kinukawa M, Minato Y, Sasada H, Kohsaka T (2017). Functional insulin-like factor 3 (INSL3) hormone-receptor system in the testes and spermatozoa of domestic ruminants and its potential as a predictor of sire fertility. Anim Sci J.

[B030] Qianmei W, Ligang Y, Chenye L, Hongzao Y, Shaoyu C (2020). Distribution of sex hormone receptors in cryptorchidism and normal tests of ziwuling black goat. Acta Vet Zootech Sin.

[B031] Qin W, Wang B, Yang L, Yuan Y, Xiong X, Li J, Yin S (2021). Molecular cloning, characterization, and function analysis of the AMH gene in Yak (Bos grunniens) Sertoli cells. Theriogenology.

[B032] Santamaria L, Martinez-Onsurbe P, Paniagua R, Nistal M (1990). Laminin, type IV collagen, and fibronectin in normal and cryptorchid human testes. An immunohistochemical study. Int J Androl.

[B033] Siu MKY, Cheng CY (2008). Extracellular matrix and its role in spermatogenesis. Adv Exp Med Biol.

[B034] Soito ICS, Favorito LA, Costa WS, Sampaio FJB, Cardoso LEM (2011). Extracellular matrix remodeling in the human gubernaculum during fetal testicular descent and in cryptorchidic children. World J Urol.

[B035] Taranta A, Teti A, Stefanini M, D’Agostino A (2000). Immediate cell signal induced by laminin in rat Sertoli cells. Matrix Biol.

[B036] Tung PS, Fritz IB (1980). Interactions of sertoli cells with myoid cells in vitro. Biol Reprod.

[B037] Verkauskas G, Malcius D, Dasevicius D, Hadziselimovic F (2019). Histopathology of unilateral cryptorchidism. Pediatr Dev Pathol.

[B038] Walker WH (2009). Molecular mechanisms of testosterone action in spermatogenesis. Steroids.

[B039] Wang R, Okamoto M, Xing X, Crawford NM (2003). Microarray analysis of the nitrate response in arabidopsis roots and shoots reveals over 1,000 rapidly responding genes and new linkages to glucose, trehalose-6-phosphate, iron, and sulfate metabolism. Plant Physiol.

[B040] Xing J, Bai Z (2018). Is testicular dysgenesis syndrome a genetic, endocrine, or environmental disease, or an unexplained reproductive disorder?. Life Sci.

[B041] Yuan L, Zhu J, Gu L, Yan Z (2015). The histologic characters of testis in aging yak. Histol Characters Testis Aging Yak.

[B042] Yuan L, Qu Y, Li C (2016). The histologic and ultrastructural characteristics of the Bactrian camel testis in cryptorchidism. Chinese J Anim Vet Ences.

[B043] Yuan L, Lu Y, Tao J, Zhang Y (2017). Comparison of histochemical and ultrastructural characteristics of extracellular matrix components in the scrotal and cryptorchid testes of the Bactrian camel. Acta Theriologica Sinica.

[B044] Yuan L, Zhang Y, Li C, Cheng X (2017). Comparison of distribution characteristics of extracellular matrix components in the testis of the Tibetan sheep and the small-tail Han sheep from plateau. Acta Anat Sin.

[B045] Yuan L, Wang H, Wang Q, Li C, Yang D (2021). INSL-3 protein expression in normal and cryptorchid testes of Ziwuling black goats. Reprod Domest Anim.

[B046] Zhao H, Nie J, Zhu X, Lu Y, Liang X, Xu H, Yang X, Zhang Y, Lu K, Lu S (2018). In vitro differentiation of spermatogonial stem cells using testicular cells from Guangxi Bama mini-pig. J Vet Sci.

[B047] Zhu J, Gu L, Yan Z, Tian D, Yuan L (2014). Morphological changes of testicular seminiferous tubules and Leyig cells of yak in different ages. Gansu Nongye Daxue Xuebao.

